# Opioid-Induced Constipation: Mechanistic Insights, Experimental Models, and Future Perspectives

**DOI:** 10.3390/biomedicines14050995

**Published:** 2026-04-27

**Authors:** Yujia Lin, Panpan Lu, Qiang Ding, Xiang Tao, Qinghai Tan, Mei Liu

**Affiliations:** Department of Gastroenterology, Tongji Hospital, Tongji Medical College, Huazhong University of Science and Technology, 1095 Jiefang Road, Wuhan 430030, China; linyujia0217@163.com (Y.L.); lu2020tj5085@126.com (P.L.); dingqiang@tjh.tjmu.edu.cn (Q.D.); 2019509088@hust.edu.cn (X.T.)

**Keywords:** opioid-induced constipation, motility, intestinal barrier, gut microbiota, animal models

## Abstract

Opioid-induced constipation (OIC) represents a prevalent adverse effect of opioid analgesics, affecting 60–90% of patients and significantly compromising quality of life. This review delineates the multifactorial pathogenesis of OIC. Peripheral μ-opioid receptor (MOR) activation suppresses enteric neuronal excitability, inhibits intestinal motility and secretion, and impairs rectoanal function. Notably, the colon appears to exhibit a distinctive lack of tolerance to opioids. Enteric glial cell activation has been implicated in neuroinflammation, while interstitial cells of Cajal show impaired pacemaker function. Central mechanisms are increasingly recognized to involve the brain–gut axis. Furthermore, opioid-induced barrier disruption, microbiota dysbiosis, and LPS/TLR4-mediated inflammation are proposed to interact and may contribute to a self-reinforcing cycle. Animal models have been instrumental in dissecting these mechanisms. However, they present limitations in reproducibility, clinical phenotype fidelity, and translational validity, particularly regarding microbiome composition and neuroimmune responses. Future research should prioritize the development of standardized, physiologically relevant animal models incorporating multi-omics approaches, and validate mechanism-based therapeutic strategies, including peripherally acting MOR antagonists and microbiota-targeted interventions, for precision management of OIC.

## 1. Introduction

Chronic pain affects approximately 20% of adults worldwide, which challenges pain management for clinicians [1,2,3]. Opioids are widely acknowledged as effective analgesics and have been designated by the World Health Organization as first-line agents for moderate to severe pain. Nevertheless, the diverse adverse effects associated with opioid administration—such as opioid use disorder (OUD), tolerance, dependence, and gastrointestinal dysfunction—warrant careful consideration [4,5]. Opioid-induced constipation (OIC) represents a common gastrointestinal side effect among opioid users, with an incidence ranging from 60% to 90% [6]. OIC is defined as a change in bowel habits and defecation patterns relative to baseline following opioid therapy [7], encompassing both new-onset constipation or exacerbation of pre-existing constipation upon opioid initiation or dose escalation. The Rome criteria underscore the temporal relationship and causal association between opioid administration and constipation onset. An Italian expert consensus further proposes that the persistence of constipation symptoms for at least two weeks constitutes one of the diagnostic criteria for OIC [8]. OIC markedly compromises patients’ quality of life, treatment adherence, and the sustainability of analgesic regimens, even becoming a major factor limiting adequate opioid analgesia. Therefore, systematic elucidation of OIC’s pathogenesis, the establishment of reliable animal models, and the exploration of effective preventive as well as therapeutic strategies for OIC are pivotal in order to optimize pain management and improve patient outcomes.

Studies have demonstrated that OIC is not a dysfunction mediated by a single pathway, but a complex pathophysiological process arising from multilevel and multistep interactions. The core mechanism initially involves opioid receptor activation, particularly of peripheral μ-opioid receptors (MORs) expressed in the enteric plexuses and smooth muscle of the intestinal wall which directly suppress intestinal motility and reduce luminal secretion [9]. More specifically, the enteric nervous system (ENS) plays a pivotal role in OIC pathogenesis, encompassing the inhibition of motor and secretory neurons, modulation of neuro-immune-epithelial crosstalk by enteric glial cells, attenuation of the rectoanal reflex, and the lack of tolerance to the inhibitory effects of opioids on gastrointestinal motility [10]. These alterations collectively contribute to diminished intestinal propulsion and defecatory dysfunction. Beyond peripheral intestinal mechanisms, both the central nervous system (CNS) and interstitial cells of Cajal (ICCs), which serve as the intestinal pacemakers, participate in opioid-mediated inhibition of gut function [11,12]. Emerging evidence also indicates that OIC development is accompanied by gut microbiota dysbiosis, wherein alterations in microbial composition and metabolites can influence enteric neural function, barrier homeostasis, and the inflammatory microenvironment. In parallel, intestinal barrier disruption and inflammatory responses may exacerbate intestinal sensory and motor abnormalities that will prompt a vicious cycle eventually [13,14]. These mechanisms collectively constitute the multidimensional, cross-system regulatory pathological basis of OIC.

Concurrent with the deepening mechanistic insights, animal models have provided critical experimental platforms for dissecting the pathological processes of OIC and evaluating interventional strategies. Currently employed OIC modeling approaches include acute and chronic opioid administration paradigms, with various delivery routes (e.g., subcutaneous, intraperitoneal). Model validity is assessed through functional indices, parameters related to intestinal secretion and absorption, and evaluations of neuromuscular function, as well as molecular and histopathological markers. Nevertheless, substantial variations persist among different models regarding stability and reproducibility. The development of animal models with enhanced translational value and the establishment of standardized, multidimensional evaluation systems represent significant challenges in current basic research on OIC.

**Figure 1:**
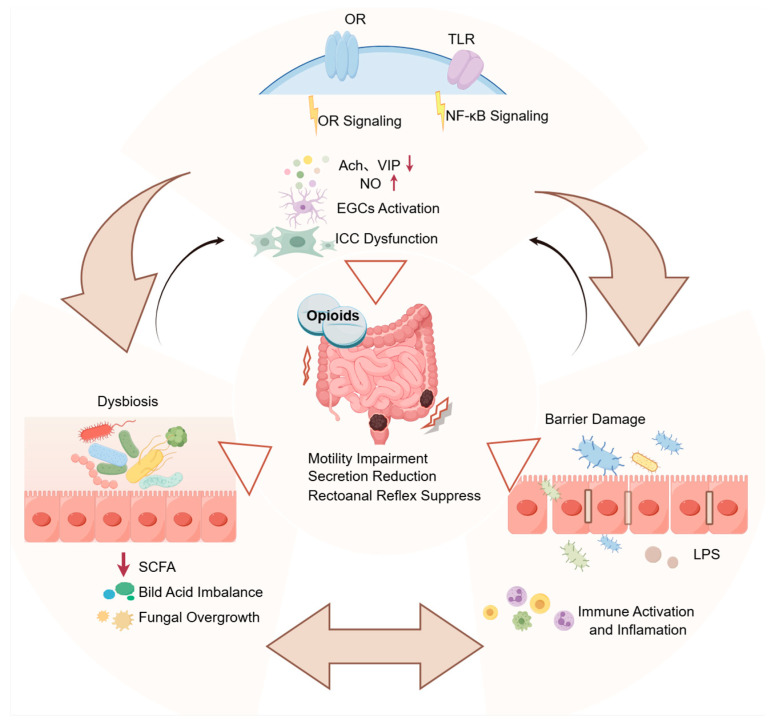
Schematic illustration of the pathophysiological mechanisms underlying opioid-induced constipation (OIC). Activation of opioid receptors disrupts enteric neurotransmitter homeostasis, characterized by reduced levels of acetylcholine (ACh) and vasoactive intestinal peptide (VIP) and increased nitric oxide (NO), thereby inhibiting excitatory motor neurons and secretomotor neurons and impairing sensory neuronal function. Concurrent activation of enteric glial cells (EGCs), together with a reduction in the number and functional impairment of interstitial cells of Cajal (ICCs), collectively attenuates propulsive peristalsis and epithelial secretion and suppresses rectoanal reflexes. In addition, opioid-induced gut dysbiosis and intestinal barrier damage, superimposed on inflammatory responses, act synergistically and reciprocally to further exacerbate impairments in intestinal motility and secretory function. Overall, these mechanisms promote both the initiation and perpetuation of OIC. OR: opioid receptor; TLR: Toll-like receptor; EGCs: enteric glial cells; ICCs: interstitial cells of Cajal; SCFAs: short-chain fatty acids; LPS: lipopolysaccharide. Created by figdraw.com.

This review discussed the principal pathogenic mechanisms of OIC (as illustrated in Figure 1), and focused on integrated analysis of critical components including opioid receptor signaling, enteric nervous system regulation, the ICC functional alterations, and gut microbiota–barrier–inflammation interactions. Additionally, we outlined the strategies of commonly applied OIC animal models as well as related indicators, and analyzed the advantages and limitations of evaluation indicators to OIC in different models. Finally, the outlook of OIC research, such as the development and standardization of animal models with enhanced physiological relevance and the optimization of clinically translatable strategies, was discussed. This work is expected to be helpful for OIC research in future.

## 2. Methods of Literature Review

### 2.1. Literature Search Strategy

This work was conducted as a structured narrative literature review that focuses on the pathophysiological mechanisms and the experimental models of OIC. Perspectives on OIC treatment, especially mechanism-informed targets rather than a comprehensive evaluation of clinical treatments, were also discussed.

Studies were searched from PubMed, Web of Science, and Scopus that were published up to December 2025, comprehensively. The search strategy combined controlled vocabulary and free-text terms, including “opioid-induced constipation,” “opioid receptors,” “μ-opioid receptor” OR “MOR,” “enteric nervous system,” “intestinal motility,” “gut barrier,” “microbiota,” and “animal models”. Boolean operators (AND/OR) were applied to optimize retrieval. In addition, the reference lists of relevant studies were manually screened to identify further eligible studies.

### 2.2. Study Selection and Eligibility Criteria

Studies were included if they met the following criteria: (i) original research articles or reviews addressing OIC mechanisms, experimental models, or therapeutic approaches; (ii) studies based on animal models; (iii) investigations providing mechanistic, functional, or clinically relevant insights into opioid-associated gastrointestinal dysfunction.

Exclusion criteria were as follows: studies focusing exclusively on non-opioid-related constipation without mechanistic relevance to opioid signaling were excluded. Conference abstracts without sufficient methodological detail and non-English publications were also excluded.

### 2.3. Data Extraction and Synthesis

Data extraction was conducted using a qualitative approach, focusing on key study characteristics including animal models, experimental design, opioid agents used, routes and duration of exposure, and principal mechanistic or functional outcomes. Given the heterogeneity in study designs, endpoints, and reporting formats, a formal meta-analysis was not performed. Instead, a narrative synthesis approach was adopted, emphasizing cross-study consistency and biological plausibility.

## 3. What Is OIC

Constipation is broadly categorized into primary (functional) constipation and secondary constipation, with the latter encompassing diverse etiologies including metabolic disorders, structural abnormalities, and medication side effects. OIC represents a distinct pharmacological subtype of secondary constipation that differs fundamentally from functional constipation in both pathophysiology and clinical management. The first consensus definition for OIC was established in 2014 by Camilleri and colleagues, who emphasized temporal causality, specifically, the emergence or exacerbation of constipation symptoms following opioid initiation [15]. Core diagnostic features included ≥2 of the following—reduced bowel movement frequency, increased straining, sensation of incomplete evacuation, or hardening of stool consistency—all measured against the patient’s pretreatment baseline. The Rome IV criteria (2016) subsequently refined this framework by formally delineating OIC from functional constipation [7,8]. The committee defined OIC as new or worsening constipation symptoms (fulfilling more than two of the six Rome criteria for functional constipation) that develop in temporal association with opioid initiation, formulation modification, or dose escalation, with loose stools rarely present in the absence of laxative therapy [7,8]. These definitions underscore three critical elements: (i) the obligatory causal relationship with opioid exposure; (ii) the change-from-baseline paradigm; and (iii) the characteristic absence of diarrhea without pharmacological intervention. These are features that collectively distinguish OIC from other constipation subtypes.

## 4. Pathogenesis of OIC

### 4.1. Activation of Opioid Receptors

Opioid receptors are seven-transmembrane G-protein-coupled receptors (GPCRs) that are widely expressed in both the central and peripheral nervous systems [16]. The classical opioid receptor family comprises three canonical subtypes—μ (MOR), κ (KOR), and δ (DOR)—together with the nociceptin/orphanin FQ receptor (NOP), which is structurally related but pharmacologically distinct [17,18]. These receptors are activated by endogenous opioid peptides, including endorphins, enkephalins, dynorphins, and nociceptin, as well as exogenous opioid ligands. These receptor systems play central roles in regulating nociception, reward and aversion, stress responses, mood, gastrointestinal motility, and neuroendocrine and immune functions [19]. Notably, Mas-related G-protein-coupled receptors (MRGPRs), such as MRGPRX1 and MRGPRX2, although implicated in pruritus and pain signaling [20,21], will not be discussed in this context, because they are not classified as opioid receptors.

Upon agonist binding, opioid receptors primarily couple to inhibitory G proteins (Gαi/o). This leads to GDP–GTP exchange on the Gα subunit, followed by dissociation of Gαi/o and Gβγ subunits, which initiate downstream signaling cascades [22,23]. The Gαi/o subunit inhibits adenylyl cyclase activity, resulting in decreased intracellular cyclic adenosine monophosphate (cAMP) levels. In parallel, Gβγ subunits modulate ion channel activity, including activation of G-protein-gated inwardly rectifying potassium (GIRK) channels and inhibition of voltage-gated calcium channels, thereby promoting membrane hyperpolarization and reducing neuronal excitability and neurotransmitter release [24,25,26,27,28].

Following activation, opioid receptors are typically phosphorylated at their intracellular serine and threonine residues within their C-terminal tails, mediated by G-protein-coupled receptor kinases (GRKs) and second-messenger-regulated kinases, such as protein kinase C (PKC). This phosphorylation facilitates β-arrestin recruitment, leading to receptor desensitization and clathrin-mediated internalization, with subsequent receptor recycling or degradation depending on cellular context [24,29].

### 4.2. Enteric Nervous System and OIC

The enteric nervous system (ENS) is capable of executing a range of essential functions independently of the CNS, including peristalsis, regulation of intestinal blood flow, and gastrointestinal secretion [30,31,32]. ENS neurons are located in the submucosal plexus within the submucosal layer and the myenteric plexus situated between the two layers of the external musculature [33]. The former regulates intestinal secretion and blood flow, whereas the latter coordinates gastrointestinal motility. Accumulating evidence indicates that opioid receptors are widely distributed within the ENS, with expression detected in the submucosal plexus, myenteric plexus, and the longitudinal muscle layer of the ileum [34].

#### 4.2.1. Myenteric Neuron-Mediated Impairment of Intestinal Motility

Colonic propulsion requires coordinated cycles of contraction and relaxation of the colonic musculature. Myenteric neurons regulate intestinal motility by releasing acetylcholine (ACh) and substance P to induce muscle contraction, and via the release of ATP/β-NAD, nitric oxide (NO), and vasoactive intestinal peptide (VIP) to facilitate muscle relaxation [35,36].

MORs and DORs represent the predominant inhibitory G proteins on intestinal neurons and are regarded as the principal mediators of opioid-induced gastrointestinal dysmotility [34,37,38]. In addition, κ-opioid receptors (KORs) are also expressed in the enteric nervous system, including myenteric neurons [34,37], where they can modulate neurotransmitter release and neuronal excitability via similar Gi/o-protein-coupled mechanisms [34,37]. However, compared with MORs, the role of KORs in regulating intestinal motility appears to be weak and remains not well characterized [34,39]. Many studies suggest that KOR activation may exert modulatory or context-dependent effects on gastrointestinal transit, potentially involving interactions with inhibitory neural pathways. Mechanistically, these opioid receptors share a common coupling with inhibitory Gi/o proteins [34]. Upon activation by opioid agonists, they dissociate into Gαi/o and Gβγ subunits, which coordinately inhibit adenylyl cyclase (AC), reduce intracellular cAMP levels, and decrease protein kinase A (PKA) activity [40]. These signaling events ultimately promote membrane hyperpolarization of enteric neurons and suppress neuronal excitability. This slow afterhyperpolarization (SAHP) reduces neuronal excitability and disrupts synaptic signal transmission [41].

Recent studies have revealed that morphine attenuates action potentials in myenteric neurons by inhibiting Na^+^ channels, thereby impairing cellular excitability and neuromuscular signal transmission in the murine intestine [42]. In parallel, activation of opioid receptors inhibits neuronal voltage-gated calcium channels (VGCCs) leading to reduced Ca^2+^ influx upon membrane depolarization and consequent suppression of neurotransmitter release [43]. A study by Iwata and colleagues suggested that the nitrergic pathways may also participate in opioid-induced effects within the gastrointestinal tract [44]. Tetrodotoxin and N-nitro-L-arginine were shown to inhibit morphine-induced ileal contractions in mice, indicating that the actions of morphine may, at least in part, be related to suppression of inhibitory nitrergic neurotransmission. In addition, Jia et al. recently demonstrated in an OIC rat model that activation of neuronal MORs in the colonic myenteric plexus induces hyperphosphorylation of α-synuclein at Ser129 (pS129-α-Syn), leading to marked downregulation of synaptic-function-related proteins (Synapsin-1, Synaptotagmin-1, VAMP-2, Syntaxin-1) and neurotransmitter synthetic enzymes (nNOS, ATPB) [45]. These changes impair synaptic vesicle trafficking and transmitter release, ultimately resulting in colonic dysmotility and constipation. This signaling pathway provides a mechanistic explanation for the deeper neuro-molecular imbalance of ENS function in chronic OIC, extending beyond transient functional inhibition.

#### 4.2.2. Secretory Neuron-Mediated Reduction in Intestinal Fluid Secretion

Submucosal secretomotor neurons stimulate electrolyte and water secretion by intestinal epithelial cells through the release of ACh and VIP from nerve terminals [35]. Ion secretion mediated by chloride channel activation is recognized as the primary driving force for intestinal secretion, involving the cystic fibrosis transmembrane conductance regulator (CFTR) and ClC-2 channels. Opioids predominantly activate DORs and MORs on secretomotor neurons, resulting in reduced ACh and VIP release, thereby decreasing intestinal epithelial Cl^−^ secretion and water transport [46,47,48]. Additionally, enteric neurotransmitters such as nitric oxide and somatostatin, as well as gastrointestinal hormones, are implicated in promoting OIC pathogenesis; inadequate activation of these signaling molecules disrupts water transport in intestinal epithelial cells [49].

#### 4.2.3. Sensory Neuron- and Smooth Muscle-Mediated Anorectal Dysfunction

Impaired rectal sensation is considered as a critical pathophysiological factor contributing to constipation. Diminished rectal sensation is closely associated with opioid use in individuals with moderate to severe pain [50]. Comprehensive anorectal physiological assessments in this study revealed that, in addition to inhibiting intestinal motility, opioid agonists act directly on intestinal sensory neurons, reducing their responsiveness to mechanical distension, such as fecal filling. This results in delayed or absent defecatory urge, depriving patients of the initial signal required to initiate bowel movements. Notably, dysfunction of the internal anal sphincter (IAS) is regarded as a contributing factor to the development of OIC [51]. Gastrointestinal sphincters, including the lower esophageal sphincter, pyloric sphincter, sphincter of Oddi, ileocecal valve sphincter, and internal and external anal sphincters, are abundantly enriched with opioid receptors (particularly MORs and DORs). Opioid agonists directly induce sustained smooth muscle contraction or increased resting tone through activation of these receptors. The IAS, composed of smooth muscle, is the primary structure responsible for maintaining resting anal pressure, and abnormal elevation of its tone significantly disrupts normal defecatory reflexes. Under opioid exposure, relaxation of the IAS triggered by rectal distension—the rectoanal inhibitory reflex—is attenuated, impeding fecal expulsion.

#### 4.2.4. Opioid-Induced Enteric Glial Cell Dysfunction

In physiological conditions, enteric glial cells (EGCs) support and nourish enteric neurons, maintain intestinal barrier integrity through the release of various trophic factors and signaling molecules, modulate neurotransmission, and participate in immune surveillance and regulation [52]. Distinct from their direct inhibitory effects on neurons, opioid agonists activate EGCs. Gao et al. demonstrated that EGCs express MORs. Morphine induces reactive glial activation, characterized by upregulation of glial fibrillary acidic protein and increased release of proinflammatory mediators. Silencing MORs partially reverses this inflammatory phenotype in EGCs, indicating a mechanistic role of glial MOR signaling in OIC pathogenesis [53]. The purinergic signaling pathway may serve as a critical downstream effector mechanism promoting EGC-mediated OIC. In the study by Bhave S et al., exposure of rat EGCs to morphine markedly increased P2X receptor activity. Notably, this effect was absent in vitro experiments, demonstrating that morphine, in vivo, acts in concert with lipopolysaccharide (LPS) stimulation from the gut to enhance ATP release from EGCs and to augment connexin 43 (Cx43)-mediated intercellular communication [54]. Glial-derived ATP can act on neuronal P2X receptors, promoting neuronal death and neuroinflammation [55,56], thereby disrupting normal intestinal motility and secretion. The discrepancy between in vivo and in vitro findings further highlights the pivotal role of intestinal microbiota and the inflammatory microenvironment in the morphine–EGC–constipation pathophysiological axis. At the neural circuit level, enteric neuronal injury combined with glial dysfunction collectively contribute to colonic migrating motor complex (CMMC) dysfunction, which relies on cholinergic transmission and the NO/cGMP pathway [57]. Additionally, morphine treatment reduces EGC secretion of glial cell line-derived neurotrophic factor (GDNF), compromising intestinal epithelial cell (IEC) barrier integrity [58]. Concomitant increases in intestinal permeability and bacterial translocation further activate TLR4, thereby establishing a vicious cycle of EGC-mediated neuroinflammation [59].

#### 4.2.5. Lack of Tolerance

Adverse gastrointestinal effects of opioids, particularly in the colon, persist regardless of the duration of opioid exposure [60]. Activated opioid receptors are usually phosphorylated by G-protein-coupled receptor kinases (GRKs), facilitating receptor interaction with β-arrestins (β-arrestin 1 and 2), followed by receptor endocytosis, intracellular sorting, and recycling to the plasma membrane [28]. This regulatory process governs opioid receptor desensitization, downregulation, and resensitization, and is closely linked to cellular responsiveness and the development of tolerance. Studies have shown that morphine-induced contraction amplitude in small-intestinal circular muscle diminishes with repeated morphine administration, whereas this attenuation is not observed in the colon, suggesting that the small intestine can develop tolerance to opioids while the colon cannot [61,62]. Previous investigations comparing morphine tolerance in murine ileum and colon have attributed this differential effect to distinct MOR–β-arrestin-2 interactions [37,63]. In contrast to the ileum, β-arrestin-2 levels in the colon do not decline under sustained opioid receptor activation, which may account for the lack of observable opioid tolerance [45]. Additionally, studies have demonstrated that β-arrestin levels remain unchanged in guinea pig and rat ileum following in vitro incubation for 4–7 days in vivo [64,65], suggesting the involvement of additional β-arrestin-independent mechanisms in the development of opioid tolerance.

### 4.3. Central Nervous System and OIC

Opioid receptors are extensively distributed throughout the CNS, with MORs, DORs, and KORs highly expressed in pain-modulatory pathways, limbic structures, and cortical regions [66]. Opioids exert analgesic effects through presynaptic inhibition of neurotransmitter release and postsynaptic hyperpolarization, leading to reduced neuronal excitability [67]. In addition, opioid receptor activation modulates reward circuitry, dopaminergic signaling, and emotional and stress-related processes within the CNS [5,68]. These central actions are associated with opioid-related adverse effects, including addiction, sedation, depression, and anxiety. Emerging research suggests that central neural circuits may contribute to the regulation of gastrointestinal function during opioid exposure. For example, Ma and colleagues described a trisynaptic pathway involving hypothalamic paraventricular nucleus glutamatergic neurons (PVN^Glu^), dorsal motor nucleus of the vagus cholinergic neurons (DMV^ACh^), and downstream intestinal motility [69]. This pathway has been proposed as one potential neural mechanism by which morphine may suppress small-intestinal propulsion.

Furthermore, chronic engagement of central reward- and stress-related circuits by opioids may influence enteric function via neuroendocrine and neuroimmune pathways, including activation of the hypothalamic–pituitary–adrenal (HPA) axis [70,71]. These central–peripheral interactions have been implicated in modulating enteric neuroinflammation and neuro-glial homeostasis, which may contribute to the persistence or exacerbation of OIC. However, the precise causal relationships and relative contributions of these CNS-mediated mechanisms remain to be fully established.

### 4.4. Interstitial Cells of Cajal and OIC

Interstitial cells of Cajal (ICCs) represent a specialized population of pacemaker cells and signal transduction stations located within the muscular layers of the gastrointestinal wall. They receive excitatory or inhibitory signals from enteric neurons (such as ACh and NO), integrate and amplify these signals, and subsequently transmit them to smooth muscle cells to initiate or suppress contractions, thereby generating intestinal slow waves and peristalsis [72]. ICC depletion or damage has been documented in various gastrointestinal motility disorders [73,74]. Opioid agonists may indirectly compromise intestinal motility through effects on ICCs. Experimental evidence demonstrates that activation of intestinal μ-opioid receptors in mice is associated with decreased c-Kit expression and ICC population [75]. Naloxone treatment restores pacemaker currents and cellular phenotypes of ICCs in the proximal colon of rabbits receiving epidural morphine infusion [76,77]. Although the precise mechanisms underlying opioid-induced ICC reduction remain elusive, it is established that ICC maintenance, survival, and proliferation depend on Ano1, c-Kit, and 5-HT signaling [78,79]. Inflammatory pathways such as LPS-TLR4/-TNF-α suppress these signals and lead to aberrant ICC phenotypes and functional impairment. Notably, TNF-α-induced ICC reduction exhibits partial reversibility [80]. Furthermore, ICCs also express neuropeptide receptors and demonstrate sensitivity to NO [81,82]. Under inflammatory conditions, oxidative stress induced by NO, which is synthesized by macrophages and smooth muscle cells, is detrimental to ICCs and may constitute a primary cause of ICC structural and functional alterations, implicating a critical regulatory role of the inflammatory microenvironment in ICC homeostasis [83,84].

Opioid exposure induces intestinal dysbiosis accompanied by increased LPS production [29], thereby activating innate immune responses and promoting M1 macrophage polarization. Studies in Ednrb-deficient mice have shown that macrophage depletion or TNF-α neutralization restores the morphology and pacemaker function of Kit-positive ICCs [85]. Concurrently, morphine attenuates the immunomodulatory capacity of intestinal epithelium-derived extracellular vesicles (EVs), further amplifying inflammatory responses [86]. Emerging evidence suggests that microbiota-targeted interventions may restore ICC population homeostasis through increased short-chain fatty acids (SCFAs) and enhanced 5-HT secretion [87]. However, the causal relationships and precise molecular mechanisms linking opioid-induced dysbiosis, immune activation, and ICC dysfunction warrant further investigation.

### 4.5. Microbiota Dysbiosis and Barrier Disruption in Gut and OIC

Chronic opioid use has been associated with intestinal barrier dysfunction, microbiota dysbiosis, and low-grade inflammation, which may collectively contribute to the development and persistence of OIC. Rather than representing a strictly linear process, these factors are increasingly considered to interact in a potentially self-reinforcing manner.

Wang et al. observed that morphine-exposed mice exhibited alterations in intestinal barrier integrity, including reduced tight junction protein expression, decreased mucus-producing goblet cells, and impaired immune responses [88]. The intestinal barrier consists of mucus layers, epithelial cells, commensal microbiota, and immune components that function cooperatively to maintain gut homeostasis. Mechanistically, opioid receptor activation and Toll-like receptor 4 (TLR4) signaling have been implicated in barrier disruption. Activation of TLR4 may induce phosphorylation of myosin light chain kinase (MLCK), leading to cytoskeletal reorganization and redistribution of tight junction proteins (e.g., occludin and ZO-1), thereby increasing intestinal permeability [59]. Additionally, TLR4 activation triggers classical downstream inflammatory signaling pathways, such as NF-κB signaling, associated with increased production of proinflammatory cytokines (e.g., TNF-α and IL-6), which may further impair barrier integrity [89]. Increased permeability may facilitate endotoxin translocation, which in turn can sustain immune activation and inflammatory responses. Inflammatory conditions have also been reported to modulate opioid receptor function. For example, intestinal inflammation has been associated with enhanced sensitivity to MOR and DOR agonists and altered receptor expression patterns, potentially amplifying opioid-induced inhibition of gastrointestinal motility [90].

Opioid-induced alterations in gut microbiota composition have been widely reported, although findings vary across studies. In animal models, opioid exposure has been associated with shifts in major bacterial phyla, including changes in the relative abundance of Firmicutes and Bacteroidetes, as well as enrichment of genera such as *Staphylococcus*, *Enterococcus*, *Fusobacterium*, and *Clostridium* [91,92]. However, inconsistencies exist, particularly regarding taxa such as Bifidobacterium, reflecting differences in experimental conditions and analytical approaches.

In addition to compositional changes, opioid exposure has been associated with alterations in microbial metabolites. For instance, reductions in short-chain fatty acids (SCFAs) have been reported in individuals receiving long-term opioid therapy [93]. SCFAs are known to influence intestinal motility, epithelial integrity, and immune regulation, partly through modulation of enteroendocrine hormones such as glucagon-like peptide-1 (GLP-1) and peptide YY (PYY) [94,95]. Experimental studies also suggest that microbiota depletion may impair enteric neuronal integrity and motility, whereas SCFA supplementation may partially restore these functions. Disturbances in bile acid metabolism have also been observed in opioid-treated models, including reduced intestinal bile acid levels [8]. Given that bile acids regulate epithelial repair and ion transport through receptors such as the farnesoid X receptor (FXR), these alterations may contribute to both barrier dysfunction and impaired intestinal secretion [96].

Overall, current evidence suggests that microbiota dysbiosis, metabolite alterations, and barrier dysfunction are interconnected processes that may collectively influence intestinal motility and inflammation. However, the temporal sequence and causal relationships among these factors remain incompletely understood. Their interactions are therefore more appropriately interpreted as a potentially self-reinforcing network rather than a definitively established linear pathway.

## 5. Experimental Models for the Study of OIC

### 5.1. Animal Models and Methodologies for OIC Construction

#### 5.1.1. Pharmacological Agents and Administration Paradigms

A range of pharmacological agents has been employed to establish animal models for OIC study. For conceptual clarity, the models discussed in this review can be broadly categorized into two groups: (i) clinically oriented OIC models, typically based on systemically administered opioids (e.g., morphine), which engage both central and peripheral pathways; and (ii) opioid-receptor-mediated constipation models, commonly using peripherally restricted agonists (e.g., loperamide), which primarily isolate gut-specific μ-opioid receptor signaling.

As summarized in Table 1, loperamide-induced models are primarily established via repeated oral administration, typically at a dose of approximately 10 mg/kg once daily for 7–14 consecutive days [97,98,99,100]. This regimen consistently produces a robust and reproducible OIC-like phenotype across studies, characterized by reduced gastrointestinal transit, decreased fecal output, and lower fecal water content. Notably, the route of administration plays a critical role in shaping the pharmacodynamic profile of loperamide. Oral administration most closely reflects its clinical use and peripheral mechanism of action, as loperamide exhibits limited blood–brain barrier permeability and primarily acts on μ-opioid receptors in the gastrointestinal tract [34,101]. In contrast, subcutaneous administration results in more rapid systemic exposure and a more pronounced inhibitory effect on intestinal motility, albeit with reduced physiological relevance. Intraperitoneal administration, however, shows greater variability and less consistent induction of stable constipation phenotypes [102]. Collectively, oral administration is the preferred approach for modeling clinically relevant OIC, whereas alternative routes may be better suited for mechanistic studies requiring stronger pharmacological effects.

Similarly, as summarized in Table 1, morphine-based OIC models exhibit distinct pharmacological and experimental characteristics. Subcutaneous injection of morphine at doses of 10 mg/kg, administered once or twice daily for 3–7 days [103,104,105], represents one of the most consistently validated paradigms for inducing a strong and sustained constipation phenotype. Compared with loperamide, morphine exerts both central and peripheral effects, resulting in more profound inhibition of gastrointestinal motility but reduced peripheral selectivity [34]. Route-dependent differences are also evident in morphine-based models. Subcutaneous administration is most commonly used due to its reliability and high efficacy in experimental settings [106], whereas oral administration provides greater translational relevance by more closely mimicking clinical exposure, albeit with increased variability due to first-pass metabolism. Intraperitoneal administration is less standardized and is often associated with transient or less reproducible effects. In addition, other delivery routes have been reported, including intravenous injection [107], intracerebroventricular administration [90], and surgically implanted pellets [54,61,108].

A key mechanistic distinction between loperamide- and morphine-based models lies in their pharmacological profiles. Loperamide functions primarily as a peripherally restricted μ-opioid receptor agonist, whereas morphine engages both central and peripheral opioid pathways. This fundamental difference has important implications for model selection, particularly when balancing mechanistic investigation with clinical translatability.

Beyond these classical agents, several other opioids and opioid-like compounds have also been used to induce constipation phenotypes in experimental systems, including codeine, fentanyl, oxycodone, and diphenoxylate. These compounds differ in receptor selectivity, central nervous system penetration, and pharmacokinetic properties, thereby influencing the magnitude, duration, and mechanistic basis of gastrointestinal effects. Oral codeine (12 mg/kg) significantly reduces defecation [109]. Subcutaneous fentanyl (1 mg/kg) and oxycodone (1 mg/kg) markedly suppress gastrointestinal transit [110,111,112]. Repeated administration of diphenoxylate (10 mg/kg for 20 days) reduces fecal water content and prolongs gastrointestinal transit time [113]. However, compared with morphine- and loperamide-based models, these paradigms exhibit lower experimental consistency and greater variability in study design, including differences in dosing regimens, administration routes, and outcome measures. Consequently, the current evidence base remains limited, with insufficient standardization for direct cross-study comparison. Details of these additional models are provided in Supplementary Table S1. Accordingly, they are not included in the primary comparative framework but may still be valuable in specific contexts, such as dissecting receptor-subtype-specific mechanisms or evaluating compound-specific effects.

**Table 1:** Evidence-based summary of OIC models.

Route	Dose Range (mg/kg)	Typical Regimen	Duration	Key Phenotype	Evidence Level ^1^	Translational Relevance	Refs
Morphine-based clinically oriented OIC models							
s.c.	3–40	10 mg/kg, once daily or single	3–7 days	Consistent reduction in GI transit and stool water content fecal output, with robust and sustained constipation phenotype	High (consistent multi-study evidence)	Moderate	[103,104,105]
p.o.	20	20 mg/kg	Single dosing	Moderate and variable effects due to first-pass metabolism and systemic variability	Moderate (limited evidence)	High	[111,113]
i.p.	0.5–10	Single or repeated dosing	1–3 days	Transient inhibition of GI motility with limited reproducibility	Moderate (variable results across studies)	Moderate	[69,114]
Loperamide-based opioid-receptor-mediated constipation models							
p.o.	5–15	10 mg/kg, once daily	1–2 weeks	Consistent reduction in GI transit, fecal output, and stool water content	High (consistent multi-study evidence)	High	[97,98,99,100]
s.c.	1–5	2 mg/kg, twice daily	1–7 days	Strong inhibition of intestinal motility, often exceeding physiological levels	Moderate (variable conditions across studies)	Low	[115,116,117,118,119]
i.p.	1–10	Single or repeated dosing	1–7 days	Variable and frequently transient effects on GI transit	Low (limited and inconsistent evidence)	Moderate	[45,114,120,121,122]

^1^ Evidence level was determined based on consistency and reproducibility across studies. Translational relevance reflects the extent to which the experimental design mimics clinical loperamide use.

#### 5.1.2. Acute Versus Chronic Modeling Strategies

OIC models can also be classified according to exposure paradigms, particularly acute versus chronic opioid administration. Acute models typically involve single or short-term dosing and are widely used to investigate the immediate effects of opioids on gastrointestinal motility and secretion. These models are experimentally convenient and enable rapid pharmacodynamic assessment; however, they may not fully capture the adaptive changes associated with long-term opioid exposure.

In contrast, chronic models—established through repeated injections, prolonged oral administration, or sustained-release systems (e.g., morphine pellets [54,61,108])—more closely recapitulate clinical scenarios involving long-term opioid therapy. These paradigms enable the investigation of tolerance development, receptor desensitization, and enteric nervous system plasticity, which are increasingly recognized as key components of OIC pathophysiology. Nevertheless, chronic models also introduce greater variability and experimental complexity, including cumulative systemic effects and increased inter-individual differences.

#### 5.1.3. Classification of OIC Experimental Models

OIC experimental models do not represent mutually exclusive categories; rather, they are more appropriately classified along multiple orthogonal dimensions based on their pharmacological and experimental characteristics.

(1) Pharmacological scope (peripheral vs. systemic action): Models induced by peripherally restricted opioid receptor agonists (e.g., loperamide) primarily target MORs in the enteric nervous system and are well suited for investigating gut-restricted mechanisms of motility inhibition and secretory dysfunction. In contrast, systemic opioid models (e.g., morphine, fentanyl, oxycodone), which involve both central and peripheral opioid signaling pathways, more closely reflect clinically relevant opioid exposure, particularly in the context of pain management.

(2) Exposure paradigm (acute vs. chronic): Models can also be distinguished according to the duration and mode of opioid administration. Acute administration models capture the immediate effects of opioids on gastrointestinal motility and secretion, whereas chronic paradigms—such as repeated dosing or sustained-release pellet implantation—facilitate the investigation of adaptive processes, including tolerance development, receptor desensitization, and enteric neuroplasticity.

Importantly, these dimensions are not mutually exclusive but intersect to define specific experimental configurations. For example, a given model may combine systemic opioid exposure with a chronic administration paradigm, thereby capturing both central involvement and long-term adaptive changes. This multidimensional classification framework provides a more flexible and conceptually robust basis for comparing OIC models and for selecting appropriate experimental strategies.

### 5.2. Evaluation Indices for OIC Animal Models

#### 5.2.1. Assessment of Intestinal Transit Function

Whole Gut Transit Test (WGTT): To assess gut transit time following opioid administration, OIC animals were fasted overnight and subsequently administered specific markers via oral gavage. Common markers include 5% Evans blue [123], phenol red [115,117], carmine [124], ink [97,125], and 5% activated carbon [126,127], which are solved in saline, 1.5% methylcellulose, and 5% or 10% gum Arabic [53,116,128,129]. The whole gut transit time is defined as the interval from gavage to the expulsion of the first colored fecal pellet, serving as a metric for overall intestinal motility.

Intestine Propulsion Test: The gastrointestinal (GI) transit time is important indicator for evaluating gut motility. Following the final drug administration, animals were fasted for 12 h before being administered ink or activated charcoal via oral gavage [130]. After approximately 30 min, the animals were humanely euthanized. A laparotomy was performed to excise the entire intestine (from the pylorus to the anus), and the distance traveled by the marker front was measured [97,127]. The GI transit rate (%) was calculated as follows: GI transit rate = 100 × (propulsion distance of activated charcoal/total intestinal length) [126]. The time interval from charcoal administration to euthanasia varied from 25 to 60 min according to different studies [111,116,125].

In addition to traditional markers, new materials such as fluorescent markers, barium sulfate and radioactive tracers are also used to assess GI transit time. For instance, FITC-dextran is utilized to quantify fluorescent signals in the supernatant of each intestinal segment through a multi-well fluorescence plate reader [65,108]. In this method, the geometric center (GC) value of fluorescence distribution is calculated, where a higher GC value indicates more rapid intestinal transit. Similarly, Grenald et al. administered milk labeled with Cr^51^ to rats and measured radioactivity in different intestinal segments via a gamma counter [131]. When utilizing barium sulfate suspension as a contrast agent to visualize intestinal transit, digital imaging devices are required [132]. Typically, animals are restrained in the prone position to visualize intestinal transit. Notably, focal length and exposure time are adjusted to guarantee the accurate percentage of the GI tract in this condition [122,133].

Colon Bead Assay: Mice were fasted overnight, after which a glass bead (typically 2–3 mm in diameter) was inserted approximately 2–3 cm into the distal colon under anesthesia. Following the procedure, the mice were allowed to recover in individual cages. GI transit time was monitored from the moment the animal regained consciousness until bead expulsion [53,115]. To facilitate the identification of the expelled bead, mice were housed in clean, bedding-free cages. The time to bead expulsion was recorded as a measure of colonic motility. Williams proposed that the maximum observation period for bead expulsion should be capped at 2 h [134].

#### 5.2.2. Measurement of Fecal Parameters

**Figure 2:**
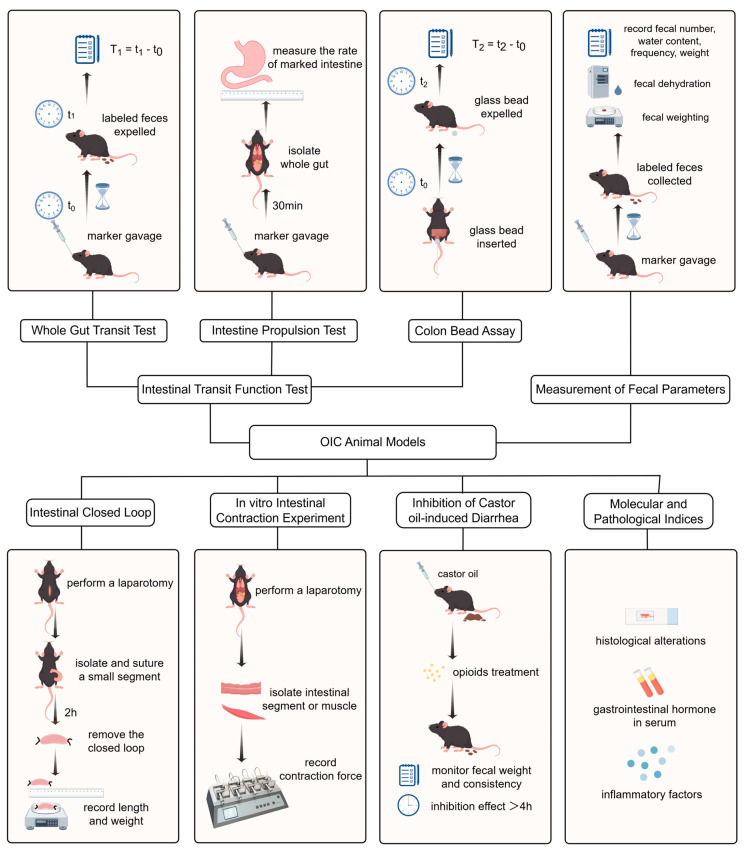
Illustration of evaluation metrics for animal models of opioid-induced constipation (OIC). Created by figdraw.com.

Stool characteristics typically evaluated include fecal number, water content, weight, and defecation frequency (as shown in Figure 2). Stool water content is calculated using the following formula:

Water content (%) = 100 × (wet weight − dry weight)/wet weight.

It is important to note that the specific measurement protocols vary across studies. For instance, Zhou reported drying feces produced between two daily doses of loperamide in a blower at 105 °C [126]. In another study, fecal properties were assessed using samples expelled within 1 h after drug administration [135]. Zhang collected feces daily and air-dried them in a laminar flow hood for 72 h. Harada et al. collected feces 16 h after codeine phosphate (CPH) treatment and dried them at 45 °C for one day [109]. Following oral gavage of activated charcoal, fecal pellets appear black due to the presence of the marker. Accordingly, in one protocol, black fecal pellets were collected for six hours following the expulsion of the first pellet [130]. Another study gathered fecal outputs from loperamide-pretreated mice over two hours and dried the samples at 65 °C overnight [118]. Across these studies, drying temperatures generally ranged from 70 °C to 80 °C overnight [135,136,137,138].

#### 5.2.3. In Vitro Intestinal Contraction Experiment

Typically, ileum and colon segments (approximately 2 cm in length) are isolated to measure intestinal contractility. The segments are ligated at both ends, connected to force transducers, and immersed in an organ chamber containing Krebs–Henseleit buffer maintained at 37 °C and aerated with 95% O_2_ and 5% CO_2_. After stabilization under modest resting tension for 60 min, isometric contractions of the isolated ileum and colon tissues are recorded [136]. Another study employed a similar organ bath experiment to measure colon contraction but utilized a distinct configuration: three high-sensitivity isometric transducers were mounted independently to measure contractile activity at the proximal, mid, and distal colon. Each transducer was attached to specific point on the colon via small stainless-steel rakes and sutures [123]. In contrast, Lin’s study focused on the contractile response of specific intestinal muscle layers rather than entire bowel segments [105]. The colonic mucosa/submucosa (M/S) and muscularis externa (ME) layers were separated via microdissection. Smooth muscle strips from the ME were then mounted in individual muscle baths and connected to isometric force transducers. Responses to various stimuli were recorded via amplifiers. Notably, tissues required washing and equilibration for 15–20 min prior to recording responses to subsequent stimulants. Furthermore, a Gastrointestinal Motility Monitoring system (GIMM) was employed in Kanemasa’s study [110]. A digital video camera tracked the propulsion of a fecal pellet from the oral to the anal end of the colon, and propulsion velocity was analyzed using GIMM software (Catamount Research and Development; St. Albans, VT, USA). Similarly, the Colon Migrating Motility Complex was assessed to record spontaneous motility across the entire colon [53]. A Logitech Pro camera captured contractile activity, and the video data were subsequently processed and analyzed.

#### 5.2.4. Intestinal Closed Loop

Mice were fasted with free access to water for 12–24 h prior to the experiment. Following anesthesia, mice were placed on a heating pad to maintain body temperature at 36 °C to 38 °C during surgery. A small abdominal incision was made to expose the small or large intestine, and a short segment was isolated and sutured at both ends to create a closed loop. The abdominal incision was then closed. After 90 min, the intestinal loops were excised, and their length and weight were measured. The length-to-weight ratio was calculated to quantify fluid secretion in the gut [121,136].

#### 5.2.5. Intestinal Single Perfusion System

Following anesthesia, experimental animals were intubated and placed on a heating pad to maintain a body temperature of 37.5 °C. Catheters were inserted into the left carotid artery to continuously monitor blood pressure and heart rate. To ensure hematocrit stability throughout the experiment, isotonic sodium bicarbonate solution and normal saline were continuously infused via the left carotid artery and left femoral artery, respectively. A midline laparotomy was performed, and the target intestinal segment (jejunum, ileum, or colon) was gently exposed and isolated while carefully preserving the mesenteric blood supply. Polyethylene catheters were inserted into both the proximal (inflow) and distal (outflow) ends of the isolated segment. Pre-warmed perfusate (pH 7.4, 37 °C; non-buffered solution containing 145.5 mM NaCl, 4.0 mM KCl, and 1.2 mM CaCl_2_) was continuously infused via perfusion pump at a constant flow rate. Effluent was collected at specific intervals. The volume difference between inflow and outflow was measured to calculate net water absorption [139].

#### 5.2.6. Inhibition on Castor Oil-Induced Diarrhea

The severity of opioid-induced constipation can also be evaluated by assessing the inhibition of castor oil-induced diarrhea. Imam et al. monitored fecal weight and consistency hourly over an 8 h period before and after administering morphine and another compound in rats [140]. Diarrhea inhibition lasting more than 4 h was classified as an indication of constipation. In a study by Varamini et al., the suppression of castor oil-induced diarrhea was used to assess the effect of morphine on fecal hydration [141]. Their results demonstrated that a single oral dose of 8 μmol/kg morphine significantly reduced castor oil-induced diarrhea.

#### 5.2.7. Molecular and Pathological Indices

Histopathological examinations are performed to evaluate histological changes in OIC models. Briefly, the procedure is as follows: colonic tissues were fixed in 4% paraformaldehyde, embedded in paraffin and sectioned. Following deparaffinization in xylene and rehydration through a graded ethanol series, sections were stained with hematoxylin and eosin (H&E) and examined under an optical microscope. Numerous studies have demonstrated distinct structural damage in the colons of various OIC models. For instance, Zhu reported overt damage in the colons of loperamide-treated mice, characterized by the detachment of mucosal epithelial cells, depletion of crypts and goblet cells, and fibrous tissue hyperplasia [97]. In addition, other studies observed increased neuronal excitability and visceral hypersensitivity in rats treated with morphine for 10 days [105]. Enzyme immunoassays revealed upregulated expression of cyclooxygenase-2 (COX-2) and nerve growth factor (NGF) in the muscularis externa of the distal colon. Zhou and colleagues collected serum from loperamide-induced mice to measure gastrointestinal hormone levels. They reported significantly increased concentrations of inhibitory hormones—endothelin-1 (ET-1), somatostatin (SS), and vasoactive intestinal peptide (VIP)—alongside reduced levels of excitatory hormones such as substance P (SP) and motilin (MTL), compared to the normal control group [126]. Qi et al. identified increased mRNA expression of major inflammatory markers, including TNFα and IL-1β [124]. Similarly, Ren’s study reported inflammatory cell infiltration in the distal colon of mice with loperamide-induced constipation [132].

### 5.3. Framework for Comparative Evaluation of OIC Models

Based on the synthesis of the experimental modeling strategies described above, a structured framework is warranted to move beyond descriptive reporting and enable systematic comparison of OIC models in terms of their physiological relevance and experimental utility. OIC models can be evaluated across the following key dimensions:

(1) Construct validity—the extent to which the model reflects opioid-receptor-mediated mechanisms, particularly MOR signaling in the enteric nervous system.

(2) Phenotype fidelity—the ability to reproduce core clinical features of OIC, including delayed gastrointestinal transit, reduced intestinal secretion, and altered neuromuscular coordination.

(3) Experimental variables—factors including route of administration (oral, subcutaneous, intravenous), dosing regimen (acute vs. chronic), and species-specific responses.

(4) Endpoint specificity—the degree to which selected assays (e.g., WGTT, bead expulsion, Ussing chamber, perfusion systems) capture distinct functional domains such as motility versus secretion.

(5) Use-case suitability—whether the model is best suited for mechanistic studies, pharmacological screening, or translational validation.

This framework enables a more systematic comparison of experimental models and highlights how methodological choices influence the interpretation and translational relevance of findings. When interpreted within this framework, distinct patterns emerge across commonly used models.

### 5.4. Assessment and Discussion

This section integrates the methodological considerations and limitations associated with commonly used experimental approaches in OIC research, with particular emphasis on how these factors influence the interpretation and translational relevance of findings.

A reduction in stool water content is widely regarded as a key indicator for successful establishment of constipation models [142]. Given the central role of ion channels and epithelial transport processes in regulating intestinal fluid balance, a range of in vivo experimental approaches has been developed to assess fluid secretion and absorption. These include closed-loop techniques, fistulous animal models, enteropooling assays, steady-state intestinal perfusion systems, and measurements of fecal water content [132,143,144]. While these models provide valuable insights into intestinal transport dynamics, they differ substantially in spatial resolution and physiological specificity. For instance, enteropooling and whole-intestine measurements offer only global assessments of fluid movement, potentially obscuring region-specific differences along the gastrointestinal tract. Moreover, compensatory absorption or secretion in distal segments may mask functional alterations occurring proximally.

In addition to these limitations in functional resolution, experimental complexity and physiological perturbations must also be considered. Surgical interventions, as required in closed-loop or perfusion-based models, may introduce confounding factors such as postoperative stress, anesthesia-related effects, and altered hemodynamics. Although some approaches attempt to mitigate these influences by allowing recovery prior to measurement, residual physiological disturbances cannot be fully excluded. Furthermore, steady-state intestinal perfusion systems demand a high level of technical expertise, including vascular catheterization and precise control of systemic parameters such as blood pressure and temperature, which may limit reproducibility across laboratories.

Assessment of gastrointestinal motility represents another central component of OIC modeling. ITTs are widely regarded as sensitive functional endpoints for detecting opioid-induced dysmotility [145]. Among available techniques, activated charcoal and dye-based assays are commonly used due to their relative simplicity and reproducibility. However, these approaches often reflect localized transit within specific intestinal segments rather than global gastrointestinal function. The colonic bead expulsion assay, while useful for evaluating distal colonic motility, may introduce mechanical stimulation that could confound interpretation. In contrast, whole gut transit assays provide a more integrative measure of gastrointestinal motility, but are subject to considerable interindividual variability.

Taken together, these observations highlight that no single experimental approach fully captures the multifaceted nature of OIC pathophysiology. Instead, different methodologies preferentially interrogate distinct functional domains, including motility, secretion, and neuromuscular coordination. Consequently, reliance on a single endpoint may lead to incomplete or potentially biased conclusions, whereas the integration of complementary assays can improve the robustness and interpretability of experimental outcomes.

The above discussion highlights the technical considerations and limitations of individual experimental approaches, while these observations can be more systematically interpreted within the comparative framework outlined in Section 5.3. When interpreted within this framework, distinct strengths and limitations emerge across commonly used OIC models.

From the perspective of construct validity, peripherally restricted agonist-based models effectively capture MOR-mediated signaling within the enteric nervous system, they do not capture central opioid effects or the integrated clinical phenotype of OIC. In contrast, morphine-based models more closely approximate the clinical condition by incorporating both central and peripheral components, although at the cost of reduced mechanistic specificity. In terms of phenotype fidelity, most models reliably reproduce delayed gastrointestinal transit and altered stool consistency; however, fewer fully recapitulate the combined features of impaired secretion, and neuromuscular dysfunction observed in clinical OIC, indicating an incomplete representation of the clinical phenotype. With regard to experimental variables, factors such as species and strain differences, route of administration, and acute versus chronic exposure paradigms significantly influence model outcomes. Notably, chronic administration strategies are more suitable for capturing adaptive processes such as tolerance and neuroplasticity, whereas acute models primarily reflect immediate pharmacodynamic effects. From the standpoint of endpoint specificity, commonly used assays differ substantially in their functional coverage. Transit-based measurements provide robust and reproducible indicators of motility but may lack resolution for segment-specific or secretory processes, while more technically demanding approaches, such as intestinal perfusion systems, offer greater mechanistic insight into fluid transport at the cost of increased experimental complexity. Finally, in terms of use-case suitability, no single model is universally optimal. Instead, model selection should be guided by the specific research objective: peripheral models are advantageous for mechanistic dissection of enteric pathways, whereas systemic and chronic paradigms offer greater translational relevance for mimicking clinical opioid exposure.

Collectively, these considerations underscore the importance of aligning model design, endpoint selection, and experimental objectives, and provide a rational basis for improving the translational relevance of future OIC studies.

## 6. Translational Perspectives and Clinical Implications in OIC

### 6.1. Translational Limitations and Optimization of Animal Models for OIC

OIC animal models have provided valuable insights into disease mechanisms, yet several limitations should be acknowledged. Firstly, establishing reproducible animal models remains challenging. Induction typically requires high doses or prolonged exposure to opioids, and outcomes may vary depending on the dose regimens, treatment duration, and route of administration. In addition, region-specific tolerance to opioid effects within the gastrointestinal tract may develop over time, contributing to variability and inconsistency in observed phenotypes [61,146]. Secondly, significant interspecies differences limit direct extrapolation to human disease. Variations in gastrointestinal anatomy, microbiota composition, and opioid receptor distribution across species constrain the translation of experimental findings. While animal models reliably recapitulate core features of OIC, such as weakened intestinal transit and reduced fecal water content, they fail to reproduce the broader spectrum of human symptoms, including abdominal discomfort and bloating.

From a translational perspective, these limitations underscore the importance of aligning model selection with specific clinical questions. For example, peripherally restricted models (e.g., loperamide-based paradigms) may be more appropriate for evaluating gut-selective mechanisms and therapies, whereas centrally penetrant opioids (e.g., morphine) better reflect the integrated pathophysiology observed in clinical OIC.

Future research should focus on optimizing model design to enhance clinical relevance. Interspecies comparisons may help identify the most appropriate models for specific clinical scenarios. In addition, the development of humanized models, such as mice grafted with human gut microbiota or organoids, will improve the predictive value of preclinical studies [93,147].

### 6.2. Mechanism-Informed Therapeutic Strategies and Clinical Implications: An Evidence-Stratified Perspective

Mechanism-based interventions and targeted drug development are increasingly recognized as important directions for advancing the prevention and management of OIC. Importantly, insights derived from experimental animal models have played a pivotal role in identifying therapeutic targets and informing clinical strategies. To provide a balanced and clinically relevant overview, therapeutic strategies are presented here according to their level of supporting evidence.

#### 6.2.1. Established Therapies with Robust Clinical Evidence

Conventional laxatives (e.g., osmotic and stimulant agents) remain the first-line therapy for OIC in many clinical settings. However, their efficacy is often limited, as they do not directly target the underlying opioid-induced mechanisms, particularly μ-opioid-receptor-mediated suppression of gastrointestinal motility [148]. Clinical studies have shown that a substantial proportion of patients experience inadequate symptom relief with laxatives alone [148,149], highlighting the need for mechanism-based therapies. Peripherally acting MOR antagonists (PAMORAs), including methylnaltrexone, naloxegol, and naldemedine, represent the most well-established mechanism-based therapies for OIC (evidence level: high; supported by randomized controlled trials and systematic reviews) Large-scale analyses have demonstrated their definitive efficacy in alleviating OIC symptoms with a low risk of serious adverse events [150]. These agents selectively antagonize MORs in the gastrointestinal tract without crossing the blood–brain barrier, thereby restoring intestinal motility while preserving central analgesia. Consistent benefits have been observed across randomized controlled trials and real-world studies, including increased spontaneous bowel movements and improved quality of life [125,126,127,128,151,152,153,154]. Nevertheless, a subset of patients remains nonresponsive to PAMORAs, highlighting the need for further investigation into optimized treatment algorithms, including combination strategies and individualized therapeutic approaches.

#### 6.2.2. Mechanistically Supported Adjunctive Strategies with Limited Clinical Evidence

Beyond direct MOR-targeted therapies, intestinal microecological modulation has emerged as a mechanistically plausible adjunctive approach, although its clinical role in OIC remains to be clearly defined (evidence level: moderate–low; limited and heterogeneous clinical data). Opioid exposure is associated with intestinal dysbiosis, which in turn impairs epithelial barrier integrity, disrupts motility, and alters neuroimmune signaling, thereby potentially exacerbating OIC [155]. Interventions aimed at restoring microbial balance, including probiotics, prebiotics, or fecal microbiota transplantation, have demonstrated beneficial effects in chronic constipation and other gastrointestinal disorders [156,157]. However, clinical evidence specifically addressing OIC is currently sparse and heterogeneous. Well-designed, adequately powered clinical trials are required to demonstrate the mechanistic relevance of microbiota-targeted interventions in this context.

#### 6.2.3. Exploratory and Emerging Pharmacological Concepts

The development of biased opioid receptor agonists represents an exploratory pharmacological strategy aimed at dissociating analgesia from gastrointestinal adverse effects (evidence level: low; primarily preclinical and early-phase clinical data). These ligands are designed to preferentially activate signaling pathways associated with analgesia while minimizing engagement of pathways implicated in gastrointestinal dysmotility and constipation [158]. Oliceridine, the first clinically approved biased agonist, has demonstrated effective analgesia in clinical studies, with a trend toward a lower incidence of gastrointestinal adverse events compared with conventional opioids [159]. However, these observations remain preliminary, and the extent to which signaling bias can translate into meaningful reductions in OIC in real-world settings remains uncertain. Further clinical validation and mechanistic refinement are required before this approach can be considered a viable strategy for OIC management.

## 7. Conclusions

Opioid-induced constipation (OIC) is a multifactorial gastrointestinal functional disorder involving the integrated effects of opioids on neural circuits (central and enteric), intestinal epithelial barrier homeostasis, immune–inflammatory pathways, and the gut microbiota–metabolite axis. Mechanistically, activation of peripheral MORs within the gastrointestinal tract suppresses the activity of excitatory motor and secretomotor neurons, attenuates propulsive motility and epithelial secretion, and contributes to anorectal dysfunction. In parallel, opioid-associated dysbiosis, barrier disruption, and inflammatory responses may interact and further exacerbate impairments in intestinal motility and secretion, thereby contributing to the persistence and progression of OIC.

Animal models have provided important experimental evidence for elucidating these interrelated mechanisms, enabling the dissection of receptor-specific signaling, epithelial and immune alterations, and microbiota-dependent effects. Nevertheless, existing models remain limited regarding opioid dosing regimens, routes of administration, segment-specific differences in gastrointestinal tolerance, and the faithful recapitulation of chronic and heterogeneous clinical phenotypes. Moreover, translational gaps persist between animal models and humans in terms of microbiome composition, immune responses, and neurogastrointestinal regulation.

Overall, current evidence supports a systems-level view of OIC pathogenesis, in which neural inhibition, intestinal barrier dysfunction, immune activation, and microbiota–metabolite disturbances interact within a potentially self-reinforcing pathological network. However, the precise causal relationships and temporal sequence among these processes remain to be fully established. Future research requires further optimization and standardization of animal models, integration of multi-omics strategies, and validation of key molecular targets in clinically relevant contexts, thereby facilitating the development of mechanism-oriented preventive and therapeutic strategies for OIC.

## Data Availability

No new data were created or analyzed in this study.

## Supplementary Materials

The following supporting information can be downloaded at: https://www.mdpi.com/article/10.3390/biomedicines14050995/s1, Table S1: Pharmacological Agents and Administration Regimens Used for OIC Animal Model Induction References [45,53,54,61,69,90,97,98,99,100,103,104,105,106,107,108,109,110,111,112,113,114,115,116,117,118,119,120,121,122,124,126,127,129,160,161,162,163,164,165,166,167,168] are cited in the Supplementary Materials.

## References

[1] Al-Khrasani M., Essmat N., Boldizsár I., Varga B.T., Chalabiani Y., Abbood S.K., Ernyey A.J., Király K., Máté A., Riba P. (2025). Do vitamins halt the COVID-19-evoked pro-inflammatory cytokines involved in the development of neuropathic pain?. Biomed. Pharmacother..

[2] Goldberg D.S., McGee S.J. (2011). Pain as a global public health priority. BMC Public Health.

[3] Brezic N., Gligorevic S., Sič A., Tseriotis V.-S., Knezevic N.N. (2026). Sexually Dimorphic Neuroimmune Pathways in Chronic Pain: A Comprehensive Systematic Review of Cellular and Molecular Mechanisms. Biomolecules.

[4] Strang J., Volkow N.D., Degenhardt L., Hickman M., Johnson K., Koob G.F., Marshall B.D.L., Tyndall M., Walsh S.L. (2020). Opioid use disorder. Nat. Rev. Dis. Primers.

[5] Essmat N., Boldizsár I., Chalabiani Y., Varga B.T., Abbood S.K., Kirchlechner-Farkas J.M., Király K., Miklya I., Gyertyán I., Tábi T. (2025). Crosstalk Between Glycinergic and N-Methyl-D-Aspartate Receptor-Mediated Glutamatergic Transmission in Behaviours Associated with Opioid Use Disorder. Int. J. Mol. Sci..

[6] Porta-Sales J., Nabal-Vicuna M., Vallano A., Espinosa J., Planas-Domingo J., Verger-Fransoy E., Julià-Torras J., Serna J., Pascual-López A., Rodríguez D. (2015). Have We Improved Pain Control in Cancer Patients? A Multicenter Study of Ambulatory and Hospitalized Cancer Patients. J. Palliat. Med..

[7] Lacy B.E., Mearin F., Chang L., Chey W.D., Lembo A.J., Simren M., Spiller R. (2016). Bowel disorders. Gastroenterology.

[8] De Giorgio R., Zucco F.M., Chiarioni G., Mercadante S., Corazziari E.S., Caraceni A., Odetti P., Giusti R., Marinangeli F., Pinto C. (2021). Management of Opioid-Induced Constipation and Bowel Dysfunction: Expert Opinion of an Italian Multidisciplinary Panel. Adv. Ther..

[9] Cash B.D. (2022). Incidence, pathophysiology, and implications of opioid-induced constipation and suggestions for patient-provider interactions. Aliment. Pharmacol. Ther..

[10] Liu M., Sheng Y., He Y., Wu S., Jin C., Shen L. (2025). Progresses in Questing for the Truth of Opioid-Related Constipation in Cancer Patients. J. Cell Mol. Med..

[11] Foong D., Zhou J., Zarrouk A., Ho V., O’Connor M.D. (2020). Understanding the Biology of Human Interstitial Cells of Cajal in Gastrointestinal Motility. Int. J. Mol. Sci..

[12] Yang H., Luo H., Li Y.H. (2019). Effects of Epidural Infusion of Morphine Combined With Small-Dose Naloxone on Gastrointestinal Interstitial Cells of Cajal in Rabbits. Eur. Rev. Med. Pharmacol. Sci..

[13] Zhang L., Meng J., Ban Y., Jalodia R., Chupikova I., Fernandez I., Brito N., Sharma U., Abreu M.T., Ramakrishnan S. (2019). Morphine Tolerance Is Attenuated in Germfree Mice and Reversed by Probiotics, Implicating the Role of Gut Microbiome. Proc. Natl. Acad. Sci. USA.

[14] Wang F., Meng J., Zhang L., Johnson T., Chen C., Roy S. (2018). Morphine Induces Changes in the Gut Microbiome and Metabolome in a Morphine Dependence Model. Sci. Rep..

[15] Gaertner J., Siemens W., Camilleri M., Davies A., Drossman D.A., Webster L.R., Becker G. (2015). Definitions and outcome measures of clinical trials regarding opioid-induced constipation: A systematic review. J. Clin. Gastroenterol..

[16] Dhaliwal A., Gupta M. (2025). Physiology, Opioid Receptor.

[17] Puls K., Schmidhammer H., Wolber G., Spetea M. (2022). Mechanistic Characterization of the Pharmacological Profile of HS-731. Molecules.

[18] Faouzi A., Varga B.R., Majumdar S. (2020). Biased Opioid Ligands. Molecules.

[19] Kiguchi N., Ding H., Cami-Kobeci G., Sukhtankar D.D., Czoty P.W., DeLoid H.B., Hsu F.C., Toll L., Husbands S.M., Ko M.C. (2019). BU10038 as a safe opioid analgesic with fewer side-effects after systemic and intrathecal administration in primates. Br. J. Anaesth..

[20] Cao C., Kang H.J., Singh I., Chen H., Zhang C., Ye W., Hayes B.W., Liu J., Gumpper R.H., Bender B.J. (2021). Structure, function and pharmacology of human itch GPCRs. Nature.

[21] Na Ayudhya C.C., Ali H. (2022). Mas-Related G Protein-Coupled Receptor-X2 and Its Role in Non-immunoglobulin E-Mediated Drug Hypersensitivity. Immunol. Allergy Clin. N. Am..

[22] Gurevich V.V., Gurevich E.V. (2019). GPCR Signaling Regulation: The Role of GRKs and Arrestins. Front. Pharmacol..

[23] Dhyani V., Gare S., Gupta R.K., Swain S., Venkatesh K.V., Giri L. (2020). GPCR mediated control of calcium dynamics: A systems perspective. Cell. Signal.

[24] Lamberts J.T., Traynor J.R. (2013). Opioid receptor interacting proteins and the control of opioid signaling. Curr. Pharm. Des..

[25] Taussig R., Iñiguez-Lluhi J.A., Gilman A.G. (1993). Inhibition of adenylyl cyclase by Gi alpha. Science.

[26] Ippolito D.L., Temkin P.A., Rogalski S.L., Chavkin C. (2002). N-terminal tyrosine residues within the potassium channel Kir3 modulate GTPase activity of Galphai. J. Biol. Chem..

[27] Roth B.L. (2019). Molecular pharmacology of metabotropic receptors targeted by neuropsychiatric drugs. Nat. Struct. Mol. Biol..

[28] Bourinet E., Soong T.W., Stea A., Snutch T.P. (1996). Determinants of the G protein-dependent opioid modulation of neuronal calcium channels. Proc. Natl. Acad. Sci. USA.

[29] Che T., Roth B.L. (2023). Molecular basis of opioid receptor signaling. Cell.

[30] Fung C., Vanden Berghe P. (2020). Functional circuits and signal processing in the enteric nervous system. Cell. Mol. Life Sci..

[31] Furness J.B. (2012). The enteric nervous system and neurogastroenterology. Nat. Rev. Gastroenterol. Hepatol..

[32] Spencer N.J., Hu H. (2020). Enteric nervous system: Sensory transduction, neural circuits and gastrointestinal motility. Nat. Rev. Gastroenterol. Hepatol..

[33] Rao M., Gershon M.D. (2018). Enteric nervous system development: What could possibly go wrong?. Nat. Rev. Neurosci..

[34] Galligan J.J., Sternini C. (2017). Insights into the role of opioid receptors in the GI tract: Experimental evidence and therapeutic relevance. Handb. Exp. Pharmacol..

[35] Brookes S.J. (2001). Classes of enteric nerve cells in the guinea-pig small intestine. Anat. Rec..

[36] Hwang S.J., Durnin L., Dwyer L., Rhee P.L., Ward S.M., Koh S.D., Sanders K.M., Mutafova-Yambolieva V.N. (2011). β-nicotinamide adenine dinucleotide is an enteric inhibitory neurotransmitter in human and nonhuman primate colons. Gastroenterology.

[37] Galligan J.J., Akbarali H.I. (2014). Molecular physiology of enteric opioid receptors. Am. J. Gastroenterol. Suppl..

[38] DiCello J.J., Carbone S.E., Saito A., Rajasekhar P., Ceredig R.A., Pham V., Valant C., Christopoulos A., Veldhuis N.A., Canals M. (2020). Mu and delta opioid receptors are coexpressed and functionally interact in the enteric nervous system of the mouse colon. Cell. Mol. Gastroenterol. Hepatol..

[39] Holzer P. (2009). Opioid receptors in the gastrointestinal tract. Regul. Pept..

[40] Chakrabarti S., Chang A., Liu N.J., Gintzler A.R. (2016). Chronic opioid treatment augments caveolin-1 scaffolding: Relevance to stimulatory μ-opioid receptor adenylyl cyclase signaling. J. Neurochem..

[41] Zhang Z., Pan Z.Z. (2010). Synaptic mechanism for functional synergism between delta- and mu-opioid receptors. J. Neurosci..

[42] Smith T.H., Grider J.R., Dewey W.L., Akbarali H.I. (2012). Morphine decreases enteric neuron excitability via inhibition of sodium channels. PLoS ONE.

[43] Weiss N., Zamponi G.W. (2021). Opioid receptor regulation of neuronal voltage-gated calcium channels. Cell. Mol. Neurobiol..

[44] Iwata H., Tsuchiya S., Nakamura T., Yano S. (2007). Morphine leads to contraction of ileal circular muscle via inhibition of nitrergic pathway. Eur. J. Pharmacol..

[45] Jia B., Zhao Y., Ren X., Zhang D., Jia H., Wang D., Wang L., Li J. (2025). Opioids induce constipation by prompting alpha-synuclein hyperphosphorylation in colonic myenteric plexus. Neurochem. Int..

[46] Fei G., Raehal K., Liu S., Qu M.H., Sun X., Wang G.D., Wang X.Y., Xia Y., Schmid C.L., Bohn L.M. (2010). Lubiprostone reverses the inhibitory action of morphine on intestinal secretion in guinea pig and mouse. J. Pharmacol. Exp. Ther..

[47] Kopic S., Corradini S., Sidani S., Murek M., Vardanyan A., Foller M., Ritter M., Geibel J.P. (2010). Ethanol inhibits gastric acid secretion through AMP-kinase activation. Cell. Physiol. Biochem..

[48] Barrett K.E. (2000). New insights into the pathogenesis of intestinal dysfunction: Secretory diarrhea and cystic fibrosis. World J. Gastroenterol..

[49] Cai T., Dong Y., Feng Z., Cai B. (2024). Ameliorative effects of the mixed aqueous extract of Aurantii Fructus Immaturus and Magnoliae Officinalis Cortex on loperamide-induced STC mice. Heliyon.

[50] Vollebregt P.F., Hooper R.L., Farmer A.D., Miller J., Knowles C.H., Scott S.M. (2020). Association between opioid usage and rectal dysfunction in constipation: A cross-sectional study of 2754 patients. Neurogastroenterol. Motil..

[51] Farmer A.D., Drewes A.M., Chiarioni G., De Giorgio R., O’Brien T., Morlion B., Tack J. (2019). Pathophysiology and management of opioid-induced constipation: European Expert Consensus Statement. United Eur. Gastroenterol. J..

[52] Gonzales J., Gulbransen B.D. (2025). The physiology of enteric glia. Annu. Rev. Physiol..

[53] Gao H., Zhang Y., Li Y., Chang H., Cheng B., Li N., Yuan W., Li S., Wang Q. (2021). μ-Opioid Receptor-Mediated Enteric Glial Activation Is Involved in Morphine-Induced Constipation. Mol. Neurobiol..

[54] Bhave S., Gade A., Kang M., Hauser K.F., Dewey W.L., Akbarali H.I. (2017). Connexin-purinergic signaling in enteric glia mediates the prolonged effect of morphine on constipation. FASEB J..

[55] Gulbransen B.D., Bashashati M., Hirota S.A., Gui X., Roberts J.A., MacDonald J.A., Muruve D.A., McKay D.M., Beck P.L., Mawe G.M. (2012). Activation of neuronal P2X7 receptor-pannexin-1 mediates death of enteric neurons during colitis. Nat. Med..

[56] Delvalle N.M., Dharshika C., Morales-Soto W., Fried D.E., Gaudette L., Gulbransen B.D. (2018). Communication between enteric neurons, glia, and nociceptors underlies the effects of tachykinins on neuroinflammation. Cell. Mol. Gastroenterol. Hepatol..

[57] Bódi N., Szalai Z., Bagyánszki M. (2019). Nitrergic enteric neurons in health and disease—Focus on Animal Models. Int. J. Mol. Sci..

[58] Bauman B.D., Meng J., Zhang L., Louiselle A., Zheng E., Banerjee S., Roy S., Segura B.J. (2017). Enteric glial-mediated intestinal barrier integrity is compromised by morphine. J. Surg. Res..

[59] Meng J., Yu H., Ma J., Wang J., Banerjee S., Charboneau R., Barke R.A., Roy S. (2013). Morphine induces acterial translocation in mice by compromising intestinal barrier function in a TLR-dependent anner. PLoS ONE.

[60] Roeland E.J., Sera C.J., Ma J.D. (2020). More opioids, more constipation? Evaluation of longitudinal total oral opioid consumption and self-reported constipation in patients with cancer. Support. Care Cancer.

[61] Ross G.R., Gabra B.H., Dewey W.L., Akbarali H.I. (2008). Morphine tolerance in mouse ileum and colon. J. Pharmacol. Exp. Ther..

[62] Akbarali H.I., Inksiar A., Dewey W.L. (2014). Site and mechanism of morphine tolerance in gastrointestinal tract. Neurogastroenterol. Motil..

[63] Muchhala K.H., Jacob J.C., Kang M., Dewey W.L., Akbarali H.I. (2021). The guts of the opioid crisis. Physiology.

[64] Kang M., Maguma H.T., Smith T.H., Ross G.R., Dewey W.L., Akbarali H.I. (2012). The role of beta-arrestin2 in the mechanism of morphine tolerance in the mouse and guinea pig gastrointestinal tract. J. Pharmacol. Exp. Ther..

[65] Duraffourd C., Kumala E., Anselmi L., Brecha N.C., Sternini C. (2014). Opioid-Induced Mitogen Activated Protein Kinase Signaling in Rat Enteric Neurons following Chronic Morphine reatment. PLoS ONE.

[66] Peng J., Sarkar S., Chang S.L. (2012). Opioid receptor expression in human brain and peripheral tissues using absolute quantitative real-time RT-PCR. Drug Alcohol. Depend..

[67] Reeves K.C., Shah N., Muñoz B., Atwood B.K. (2022). Opioid receptor-mediated regulation of neurotransmission in brain. Front. Mol. Neurosci..

[68] Zhang J.J., Song C.G., Dai J.M., Li L., Yang X.M., Chen Z.N. (2022). Mechanism of opioid addiction and its intervention therapy: Focusing on the reward circuitry and mu-opioid receptor. MedComm.

[69] Ma J., Peng X., Zhang M., Gao W., Yang X., Wang Z., Chai X., Zhang Z., Wang S., Cao P. (2026). A brain-to-small intestine circuit mediates morphine-induced constipation in male mice. Nat. Commun..

[70] Haroutounian S. (2018). Postoperative opioids, endocrine changes, and immunosuppression. Pain. Rep..

[71] Kotlińska-Lemieszek A., Żylicz Z. (2022). Less well-known consequences of long-term opioid use: A comprehensive review. Drug Des. Devel. Ther..

[72] Huizinga J.D., Hussain A., Chen J.H. (2021). Interstitial cells of Cajal and human colon motility in health and disease. Am. J. Physiol. Gastrointest. Liver Physiol..

[73] Kashyap P., Gomez-Pinilla P.J., Pozo M.J., Cima R.R., Dozois E.J., Larson D.W., Ordog T., Gibbons S.J., Farrugia G. (2011). Immunoreactivity for Ano1 detects depletion of Kit-positive interstitial cells of Cajal in patients with slow transit constipation. Neurogastroenterol. Motil..

[74] Wedel T., Spiegler J., Soellner S., Roblick U.J., Schiedeck T.H.K., Bruch H.-P., Krammer H.-J. (2002). Enteric nerves and interstitial cells of Cajal are altered in patients with slow-transit constipation and megacolon. Gastroenterology.

[75] Cheng S., Li B., Ding Y., Hou B., Hung W., He J., Jiang Y., Zhang Y., Man C. (2024). The probiotic fermented milk of Lacticaseibacillus paracasei JY062 and Lactobacillus gasseri JM1 alleviates constipation via improving gastrointestinal motility and gut microbiota. J. Dairy. Sci..

[76] Yang H., Wei F., Luo H., Li L. (2023). Effects of epidural morphine combined with low-dose naloxone on the morphology and electrophysiology of intestinal interstitial-cells of Cajal. Asian J. Surg..

[77] Yang H., Jin X.J., Luo H., Li Y.H. (2020). Effects of Morphine on Interstitial Cells of Cajal in Rabbit Colon and Small Intestinal Transit: An Experimental Study. Curr. Mol. Med..

[78] Farrugia G. (2008). Interstitial cells of Cajal in health and disease. Neurogastroenterol. Motil..

[79] Liu H.N., Ohya S., Nishizawa Y., Sawamura K., Iino S., Syed M.M., Goto K., Imaizumi Y., Nakayama S. (2011). Serotonin augments gut pacemaker activity via 5-HT3 receptors. PLoS ONE.

[80] Torihashi S., Nishi K., Tokutomi Y., Nishi T., Ward S.M., Sanders K.M. (1999). Blockade of Kit Signaling Induces Transdifferentiation of Interstitial Cells of Cajal to a Smooth Muscle Phenotype. Gastroenterology.

[81] Avetisyan M., Schill E.M., Heuckeroth R.O. (2015). Building a second brain in the bowel. J. Clin. Investig..

[82] Pasternak A., Szura M., Gil K., Matyja A. (2016). Interstitial cells of Cajal: Systematic review. Folia Morphol..

[83] Miquel J., Fleming J.E., Johnson J.E., Harman D., Walford R., Miquel J. (1986). Theoretical and experimental support for an oxygen radical–mitochondrial damage hypothesis of cell aging. Free Radicals, Aging and Degenerative Diseases.

[84] Kaji N., Horiguchi K., Iino S., Nakayama S., Ohwada T., Otani Y., Firman M., Sanders K., Ozaki H., Hori M. (2016). Nitric oxide-induced oxidative stress impairs pacemaker function of murine interstitial cells of Cajal during inflammation. Pharmacol. Res..

[85] Chen X., Meng X., Zhang H., Feng C., Wang B., Li N., Abdullahi K.M., Wu X., Yang J., Li Z. (2020). Intestinal Proinflammatory Macrophages Induce a Phenotypic Switch in Interstitial Cells of Cajal. J. Clin. Investig..

[86] Zhang Y., Yan Y., Meng J., Girotra M., Ramakrishnan S., Roy S. (2021). Immune Modulation Mediated by Extracellular Vesicles of Intestinal Organoids Is Disrupted by Opioids. Mucosal Immunol..

[87] Cheng S., Li H., Ding Y., Huo J., Zheng Y., Jiang Y., Zhang Y., Man C. (2023). The Probiotic Combination of Lacticaseibacillus Paracasei JY062 and lactobacillus gasseri JM1 Alleviates Gastrointestinal Motility Disorder via Improving Gut Microbiota. Nutrients.

[88] Wang F., Meng J., Zhang L., Roy S. (2020). Opioid use potentiates the virulence of hospital-acquired infection, increases systemic bacterial dissemination and exacerbates gut dysbiosis in a murine model of *Citrobacter rodentium* infection. Gut Microbes.

[89] Watkins L.R., Hutchinson M.R., Rice K.C., Maier S.F. (2009). Opioid-Induced Glial Activation: Improving the Clinical Efficacy of Opioids by Targeting Glia. Trends Pharmacol. Sci..

[90] Zhang Z., Zhao Y., Gou D., Li P., Wang H., Li Y., Li C., Niu Z., Yang T., Zhou L. (2025). Peripheral inflammation enhances opioid-induced gastrointestinal motility inhibition via up-regulating spinal mu opioid receptor. Toxicol. Appl. Pharmacol..

[91] Acy B.E., Cangemi D.J. (2024). Opioids and the Gastrointestinal Tract: The Role of Peripherally Active µ-Opioid Receptor Antagonists in Modulating Intestinal Permeability. Am. J. Gastroenterol..

[92] Kang M., Mischel R.A., Bhave S., Komla E., Cho A., Huang C., Dewey W.L., Akbarali H.I. (2017). The effect of gut microbiome on tolerance to morphine mediated antinociception in mice. Sci. Rep..

[93] Cruz-Lebrón A., Johnson R., Mazahery C., Troyer Z., Joussef-Piña S., Quiñones-Mateu M.E., Strauch C.M., Hazen S.L., Levine A.D. (2021). Chronic opioid use modulates human enteric microbiota and intestinal barrier integrity. Gut Microbes.

[94] Martin-Gallausiaux C., Marinelli L., Blottière H.M., Larraufie P., Lapaque N. (2021). SCFA: Mechanisms and functional importance in the gut. Proc. Nutr. Soc..

[95] Gribble F.M., Reimann F. (2019). Function and mechanisms of enteroendocrine cells and gut hormones in metabolism. Nat. Rev. Endocrinol..

[96] Keely S.J., Urso A., Ilyaskin A.V., Korbmacher C., Bunnett N.W., Poole D.P., Carbone S.E. (2022). Contributions of bile acids to gastrointestinal physiology as receptor agonists and modifiers of ion channels. Am. J. Physiol. Gastrointest. Liver Physiol..

[97] Zhu S., Yan M., Feng Y., Yin J., Jiang S., Guan Y., Gao B. (2024). Extraction of soluble dietary fiber from sunflower receptacles (*Helianthus annuus* L.) and its alleviating effect on constipation in mice. Nutrients.

[98] Yang W., Gao X., Lin J., Liu L., Peng L., Sheng J., Xu K., Tian Y. (2024). Water-insoluble dietary fiber from walnut relieves constipation through Limosilactobacillus reuteri-mediated serotonergic synapse and neuroactive ligand-receptor pathways. Int. J. Biol. Macromol..

[99] Wu T., Yang M., Jin L., Yu H., Huang H., Wu Y., Li B., Tu Y., Wan X., Liu J. (2025). Theaflavin-3,3′-digallate (TF3) attenuated constipation by promoting gastrointestinal motility and modulating the gut microbiota: A comparative study of TF3 and the anti-constipation drug mosapride in mice. Food Chem..

[100] Li M., Li Y., Cao Z., Zhang Z., Huang Y., Ming K., Cui T., Tang X., Zhang B., Deng L. (2026). Qingtong treatment principle alleviates opioid-induced constipation by regulating bile acid homeostasis via an FXR-dependent manner. J. Ethnopharmacol..

[101] MacDonald R., Heiner J., Villarreal J., Strote J. (2015). Loperamide dependence and abuse. BMJ Case Rep..

[102] Narita Y., Fukumoto K., Fukunaga M., Kondo Y., Ishitsuka Y., Jono H., Irie T., Saito H., Kadowaki D., Hirata S. (2020). Comparative Study of Constipation Exacerbation by Potassium Binders Using a Loperamide-Induced Constipation Model. Int. J. Mol. Sci..

[103] Matsumoto K., Umemoto H., Mori T., Akatsu R., Saito S., Tashima K., Shibasaki M., Kato S., Suzuki T., Horie S. (2016). Differences in the morphine-induced inhibition of small and large intestinal transit: Involvement of central and peripheral μ-opioid receptors in mice. Eur. J. Pharmacol..

[104] Tonello R., Rigo F., Gewehr C., Trevisan G., Pereira E.M.R., Gomez M.V., Ferreira J. (2014). Action of Phα1β, a peptide from the venom of the spider *Phoneutria nigriventer*, on the analgesic and adverse effects caused by morphine in mice. J. Pain.

[105] Lin Y.M., Tang Y., Fu Y., Hegde S., Shi D.W., Huang L.Y.M., Shi X.Z. (2021). An opioid receptor-independent mechanism underlies motility dysfunction and visceral hyperalgesia in opioid-induced bowel dysfunction. Am. J. Physiol. Gastrointest. Liver Physiol..

[106] Young A., Viswanath A., Kalladka M., Khan J., Eliav E., Diehl S.R. (2018). Mouse model demonstrates strain differences in susceptibility to opioid side effects. Neurosci. Lett..

[107] Wang Y., Liu X., Wang D., Yang J., Zhao L., Yu J., Wang R. (2015). Endomorphin-1 analogues (MELs) penetrate the blood-brain barrier and exhibit good analgesic effects with minimal side effects. Neuropharmacology.

[108] Gade A.R., Kang M., Khan F., Grider J.R., Damaj M.I., Dewey W.L., Akbarali H.I. (2016). Enhanced Sensitivity of α3β4 Nicotinic Receptors in Enteric Neurons after Long-Term Morphine: Implication for Opioid-Induced Constipation. J. Pharmacol. Exp. Ther..

[109] Harada Y., Iizuka S., Saegusa Y., Mogami S., Fujitsuka N., Hattori T. (2017). Mashiningan Improves Opioid-Induced Constipation in Rats by Activating Cystic Fibrosis Transmembrane Conductance Regulator Chloride Channel. J. Pharmacol. Exp. Ther..

[110] Kanemasa T., Koike K., Arai T., Ono H., Horita N., Chiba H., Nakamura A., Morioka Y., Kihara T., Hasegawa M. (2019). Pharmacologic effects of naldemedine, a peripherally acting μ-opioid receptor antagonist, in in vitro and in vivo models of opioid-induced constipation. Neurogastroenterol. Motil..

[111] Costanzini A., Ruzza C., Neto J.A., Sturaro C., Malfacini D., Sternini C., De Giorgio R., Calò G. (2021). Pharmacological characterization of naloxegol: In vitro and in vivo studies. Eur. J. Pharmacol..

[112] Kanemasa T., Koike K., Takase K., Arai T., Nakamura A., Morioka Y., Hasegawa M. (2020). Pharmacological Profile of Naldemedine, a Peripherally Acting μ-Opioid Receptor Antagonist: Comparison with Naloxone and Naloxegol. J. Pharmacol. Exp. Ther..

[113] Zhang N., Guo D., Guo N., Yang D., Yan H., Yao J., Xiao H., Shao M., Guan Y., Zhang G. (2024). Integration of UPLC-MS/MS-based metabolomics and desorption electrospray ionization-mass spectrometry imaging reveals that Shouhui Tongbian Capsule alleviates slow transit constipation by regulating bile acid metabolism. J. Chromatogr. B.

[114] Wasilewski A., Lesniak A., Bujalska-Zadrozny M., Sadowski B., Fichna J., Sacharczuk M. (2016). The effect of opioid agonists and antagonists on gastrointestinal motility in mice selected for high and low swim stress-induced analgesia. Neurogastroenterol. Motil..

[115] Zhang Y., Lu T., Meng Y., Maisiyiti A., Dong Y., Li S., Chen Y., Yin J., Chen J.D.Z. (2021). Auricular Vagal Nerve Stimulation Improves Constipation by Enhancing Colon Motility via the Central-Vagal Efferent Pathway in Opioid-Induced Constipated Rats. Neuromodulation.

[116] Li C., Nie S.P., Zhu K.X., Xiong T., Li C., Gong J., Xie M.-Y. (2015). Effect of Lactobacillus plantarum NCU116 on loperamide-induced constipation in mice. Int. J. Food Sci. Nutr..

[117] Wang X., Yang B., Yin J., Wei W., Chen J.D.Z. (2019). Electroacupuncture via chronically implanted electrodes improves gastrointestinal motility by balancing sympathovagal activities in a rat model of constipation. Am. J. Physiol. Gastrointest. Liver Physiol..

[118] Pustovit R.V., Itomi Y., Ringuet M., Diwakarla S., Chai X.-Y., McQuade R.M., Tsukimi Y., Furness J.B. (2019). Muscarinic receptor 1 allosteric modulators stimulate colorectal emptying in dog, mouse and rat and resolve constipation. Neurogastroenterol. Motil..

[119] Okada M., Itoh K., Kitakoji H., Imai K. (2025). Autonomic effects of electroacupuncture in opioid-induced constipation in male rats. Physiol. Rep..

[120] Zhao Y., Luo H., Ren X., Jia B., Li J., Wang L., Li J. (2024). The P2Y1 receptor in the colonic myenteric plexus of rats and its correlation with opioid-induced constipation. BMC Gastroenterol..

[121] Cil O., Phuan P.W., Son J.H., Zhu J.S., Ku C.K., Tabib N.A., Teuthorn A.P., Ferrera L., Zachos N.C., Lin R. (2017). Phenylquinoxalinone CFTR activator as prosecretory therapy for constipation. Transl. Res..

[122] Vera G., Girón R., Martín-Fontelles M.I., Abalo R. (2019). Radiographic dose-dependency study of loperamide effects on gastrointestinal motor function in the rat. Temporal relationship with nausea-like behavior. Neurogastroenterol. Motil..

[123] Beckett E.A.H., Staikopoulos V., Hutchinson M.R. (2018). Differential effect of morphine on gastrointestinal transit, colonic contractions and nerve-evoked relaxations in Toll-Like Receptor deficient mice. Sci. Rep..

[124] Qi B., Zhang Y., Ren D., Qin X., Wang N., Yang X. (2023). Fu Brick Tea Alleviates Constipation via Regulating the Aquaporins-Mediated Water Transport System in Association with Gut Microbiota. J. Agric. Food Chem..

[125] Li H., Xiao H.Y., Yuan L.P., Yan B., Pan Y., Tian P.P., Zhang W.J. (2023). Protective effect of L-pipecolic acid on constipation in C57BL/6 mice based on gut microbiome and serum metabolomic. BMC Microbiol..

[126] Zhou X., Chen Y., Ma X., Yu Y., Yu X., Chen X., Suo H. (2022). Efficacy of Bacillus coagulans BC01 on loperamide hydrochloride-induced constipation model in Kunming mice. Front. Nutr..

[127] Sun Y., Yan C., Jin S., Zhao J., Li G. (2020). Curative Effect and Mechanism of Guiren Runchang Granules on Morphine-Induced Slow Transit Constipation in Mice. Evid. Based Complement. Altern. Med..

[128] Sebai H., Rtibi K., Selmi S., Jridi M., Baltia R., Marzouki L. (2019). Modulating and opposite actions of two aqueous extracts prepared from Cinnamomum cassia L. bark and Quercus ilex L. on the gastrointestinal tract in rats. RSC Adv..

[129] Chen Z., Lin S.S., Jiang Y., Liu L., Jiang J., Chen S., Tong Y., Wang P. (2019). Effects of Bread Yeast Cell Wall Beta-Glucans on Mice with Loperamide-Induced Constipation. J. Med. Food.

[130] Deng M., Ye J., Zhang R., Zhang S., Dong L., Su D., Zhang M., Huang F. (2024). Shatianyu (*Citrus grandis* L. Osbeck) whole fruit alleviated loperamide-induced constipation via enhancing gut microbiota-mediated intestinal serotonin secretion and mucosal barrier homeostasis. Food Funct..

[131] Grenald S.A., Young M.A., Wang Y., Ossipov M.H., Ibrahim M.M., Largent-Milnes T.M., Vanderah T.W. (2017). Synergistic attenuation of chronic pain using mu opioid and cannabinoid receptor 2 agonists. Neuropharmacology.

[132] Ren X., Liu L., Gamallat Y., Zhang B., Xin Y. (2017). Enteromorpha and polysaccharides from enteromorpha ameliorate loperamide-induced constipation in mice. Biomed. Pharmacother..

[133] Bagues A., Girón R., Abalo R., Goicoechea C., Martín-Fontelles M.I., Sánchez-Robles E.M. (2022). Short-term stress significantly decreases morphine analgesia in trigeminal but not in spinal innervated areas in rats. Behav. Brain Res..

[134] Williams D.A., Zheng Y., David B.G., Yuan Y., Zaidi S.A., Stevens D.L., Scoggins K.L., Selley D.E., Dewey W.L., Akbarali H.I. (2016). 6β-N-Heterocyclic Substituted Naltrexamine Derivative BNAP: A Peripherally Selective Mixed MOR/KOR Ligand. ACS Chem. Neurosci..

[135] Ayari A., Dakhli N., Jedidi S., Sammari H., Arrari F., Sebai H. (2025). Laxative and purgative actions of phytoactive compounds from beetroot juice against loperamide-induced constipation, oxidative stress, and inflammation in rats. Neurogastroenterol. Motil..

[136] Cil O., Phuan P.W., Lee S., Tan J., Haggie P.M., Levin M.H., Sun L., Thiagarajah J.R., Ma T., Verkman A.S. (2016). CFTR activator increases intestinal fluid secretion and normalizes stool output in a mouse model of constipation. Cell. Mol. Gastroenterol. Hepatol..

[137] Zhang K., Luan G., Liu W., Shen F., Jiang M., Bai G. (2025). Ligustilide improves functional constipation by non-covalently activating TRPA1 in colon tissue. J. Ethnopharmacol..

[138] Xu L., Qiu B., Ba F., Zhang S., Han S., Chen H., Wu Y., Gao W., Xie S., Chen Y. (2024). Synergistic effects of Ligilactobacillus salivarius Li01 and psyllium husk prevent mice from developing loperamide-induced constipation. Food Funct..

[139] Fan W., Tan Q. (2023). Application of the steady-state intestinal perfusion system in measuring intestinal fluid absorption and bicarbonate secretion in vivo. Front. Physiol..

[140] Imam M.Z., Kuo A., Ghassabian S., Cai Y., Qin Y., Li T., Smith M.T. (2023). CYX-5, a G-protein biassed MOP receptor agonist, DOP receptor antagonist and KOP receptor agonist, evokes constipation but not respiratory depression relative to morphine in rats. Pharmacol. Rep..

[141] Varamini P., Mansfeld F.M., Blanchfield J.T., Wyse B.D., Smith M.T., Toth I. (2012). Synthesis and Biological Evaluation of an Orally Active Glycosylated Endomorphin-1. J. Med. Chem..

[142] Longstreth G.F., Thompson W.G., Chey W.D., Houghton L.A., Mearin F., Spiller R.C. (2006). Functional bowel disorders. Gastroenterology.

[143] Mourad F.H. (2004). Animal and human models for studying effects of drugs on intestinal fluid transport in vivo. J. Pharmacol. Toxicol. Methods.

[144] Naqvi S., Rehman N.U., Azhar I., Palla A. (2024). Unraveling the multi-faceted role of *Rosmarinus officinalis* L. (rosemary) and diosmetin in managing gut motility. J. Ethnopharmacol..

[145] Al-Saffar A., Takemi S., Saaed H.K., Sakata I., Sakai T. (2019). Utility of animal gastrointestinal motility and transit models in functional gastrointestinal disorders. Best Pract. Res. Clin. Gastroenterol..

[146] Essmat N., Karádi D.Á., Zádor F., Király K., Fürst S., Al-Khrasani M. (2023). Insights into the Current and Possible Future Use of Opioid Antagonists in Relation to Opioid-Induced Constipation and Dysbiosis. Molecules.

[147] Gong Z., Xue Q., Luo Y., Yu B., Hua B., Liu Z. (2024). The interplay between the microbiota and opioid in the treatment of neuropathic pain. Front. Microbiol..

[148] Emmanuel A., Johnson M., McSkimming P., Dickerson S. (2017). Laxatives Do Not Improve Symptoms of Opioid-Induced Constipation: Results of a Patient Survey. Pain Med..

[149] Coyne K.S., LoCasale R.J., Datto C.J., Sexton C.C., Yeomans K., Tack J. (2014). Opioid-induced constipation in patients with chronic noncancer pain in the USA, Canada, Germany, and the UK: Descriptive analysis of baseline patient-reported outcomes and retrospective chart review. Clinicoecon. Outcomes Res..

[150] Kistemaker K., Sijani F., Brinkman D., de Graeff A., Burchell G., Steegers M., van Zuylen L. (2024). Pharmacological prevention and treatment of opioid-induced constipation in cancer patients: A systematic review and meta-analysis. Cancer Treat. Rev..

[151] Hale M., Wild J., Reddy J., Yamada T., Arjona Ferreira J.C. (2017). Naldemedine versus placebo for opioid-induced constipation (COMPOSE-1 and COMPOSE-2): Two multicentre, phase 3, double-blind, randomised, parallel-group trials. Lancet Gastroenterol. Hepatol..

[152] Webster L.R., Nalamachu S., Morlion B., Reddy J., Baba Y., Yamada T., Ferreira J.C.A. (2018). Long-term use of naldemedine in the treatment of opioid-induced constipation in patients with chronic noncancer pain: A randomized, double-blind, placebo-controlled phase 3 study. Pain.

[153] Webster L., Dhar S., Eldon M., Masuoka L., Lappalainen J., Sostek M. (2013). A phase 2, double-blind, randomized, placebo-controlled, dose-escalation study to evaluate the efficacy, safety, and tolerability of naloxegol in patients with opioid-induced constipation. Pain.

[154] Tack J., Lappalainen J., Diva U., Tummala R., Sostek M. (2015). Efficacy and safety of naloxegol in patients with opioid-induced constipation and laxative-inadequate response. United Eur. Gastroenterol. J..

[155] Coluzzi F., Scerpa M.S., Loffredo C., Borro M., Pergolizzi J.V., LeQuang J.A., Alessandri E., Simmaco M., Rocco M. (2024). Opioid Use and Gut Dysbiosis in Cancer Pain Patients. Int. J. Mol. Sci..

[156] Chen C., Liao J., Xia Y., Liu X., Jones R., Haran J., McCormick B., Sampson T.R., Alam A., Ye K. (2022). Gut microbiota regulate alzheimer’s disease pathologies andcognitive disorders via PUFA-associated neuroinflammation. Gut.

[157] Saviano A., Brigida M., Migneco G., Gunawardena C., Zanza C., Candelli M., Franceschi F., Ojetti V. (2021). Lactobacillus Reuteri DSM 17938 *(Limosilactobacillus reuteri)* in Diarrhea and Constipation: Two Sides of the Same Coin?. Medicina.

[158] Azzam A.A.H., McDonald J., Lambert D.G. (2019). Hot Topics in Opioid Pharmacology: Mixed and Biased Opioids. Br. J. Anaesth..

[159] Markham A. (2020). Oliceridine: First approval. Drugs.

[160] Lu Y., Zhou X., Wu Y., Cui Q., Tian X., Yi H., Gong P., Zhang L. (2024). Metabolites 13,14-Dihydro-15-keto-PGE2 Participates in Bifidobacterium animalis F1-7 to Alleviate Opioid-Induced Constipation by 5-HT Pathway. Mol. Nutr. Food Res..

[161] Beltran N.M., Garcia T.I., Naime G.M., Minervini V., Serafine K.M. (2025). The effects of high fat diet consumption on morphine-induced constipation. Behav. Pharmacol..

[162] Tucci P., Palmery M., Piccolotti P., Pimpinella G., Valeri P., Romanelli L. (2008). Counteracting effect of papaverine on morphine inhibition of gastrointestinal transit in mice. Neurogastroenterol. Motil..

[163] Suzuki T., Sawada T., Kawai K., Ishihara Y. (2018). Pharmacological profile of TAN-452, a novel peripherally acting opioid receptor antagonist for the treatment of opioid-induced bowel syndromes. Life Sci..

[164] Matwyshyn G.A., Bhalla S., Gulati A. (2006). Endothelin ETA receptor blockade potentiates morphine analgesia but does not affect gastrointestinal transit in mice. Eur. J. Pharmacol..

[165] Yuan Y., Elbegdorj O., Chen J., Akubathini S.K., Zhang F., Stevens D.L., Beletskaya I.O., Scoggins K.L., Zhang Z., Gerk P.M. (2012). Design, synthesis, and biological evaluation of 17-cyclopropylmethyl-3,14β-dihydroxy-4,5α-epoxy-6β-[(4’-pyridyl)carboxamido]morphinan derivatives as peripheral selective μ opioid receptor Agents. J. Med. Chem..

[166] Farzi A., Halicka J., Mayerhofer R., Fröhlich E.E., Tatzl E., Holzer P. (2015). Toll-like receptor 4 contributes to the inhibitory effect of morphine on colonic motility in vitro and in vivo. Sci. Rep..

[167] Duron D.I., Hanak F., Streicher J.M. (2020). Daily intermittent fasting in mice enhances morphine-induced antinociception while mitigating reward, tolerance, and constipation. Pain.

[168] Wang C., Yao S., Tian B., Zhao Z., Wang Y., Li X., Li X., Huang Q. (2025). Fluoxetine enhances the treatment of depression linked to opioid-induced constipation in mice by influencing the metabolomic profile. Neurosci. Res..

